# Estimation of Yield, Photosynthetic Rate, Biochemical, and Nutritional Content of Red Leaf Lettuce (*Lactuca sativa* L.) Grown in Organic Substrates

**DOI:** 10.3390/plants10061220

**Published:** 2021-06-15

**Authors:** Md. Dulal Sarkar, Md. Jahedur Rahman, Jasim Uddain, Md. Quamruzzaman, Md. Obyedul Kalam Azad, Md. Hafizur Rahman, Md. Jahirul Islam, Mohammed Saifur Rahman, Ki-Young Choi, Most Tahera Naznin

**Affiliations:** 1 Department of Horticulture, Sher-e-Bangla Agricultural University, Dhaka 1207, Bangladesh; jrahman04@yahoo.com (M.J.R.); Uddain.jasim@gmail.com (J.U.); 2 Tasmania Institute of Agriculture, University of Tasmania, Launceston 7250, Australia; md.quamruzzaman@utas.edu.au; 3 Department of Bio-Health Convergence, College of Biomedical Science, Kangwon National University, Chuncheon 24341, Korea; azadokalam@gmail.com (M.O.K.A.); hafizknu94@gmail.com (M.H.R.); jahirulislam213@gmail.com (M.J.I.); 4 Physiology and Sugar Chemistry Division, Bangladesh Sugar Crop Research Institute, Ishurdi, Pabna 6620, Bangladesh; 5 Department of Biosystems and Technology, Swedish University of Agricultural Sciences, P.O. Box 103, SE23053 Alnarp, Sweden; morn0004@stud.slu.se; 6 Department of Controlled Agriculture, College of Agriculture and Life Sciences, Kangwon National University, Chuncheon 24341, Korea

**Keywords:** secondary metabolites, nutrient content, organic substrates, colored lettuce

## Abstract

This study aimed to evaluate the effect of organic substrates on the growth yield, photosynthetic response, and nutritional profile of red leaf lettuce grown in different compositions of cocopeat (CP), sawdust (SD), and rice husk (RH). The result showed that the properties of substrates were influenced variably by their mixing ratios. The highest water holding capacity and moisture content were found in CP, and it provided the preferable pH, electrical conductivity, bulk density, and air-filled porosity in association with other categories of the substrate. Cocopeat-based media provides ample microclimate conditions in the root region of plants and increased their height, number of leaves, and fresh biomass components. The utmost dry biomass of plant parts also remarkably increased in CP; L*, a*, and b* chromaticity of leaves remained unchanged. The maximum chlorophyll content was attained in CP substrate, except for chlorophyll a/b, which was higher in RH. The net photosynthetic rate (PN), transpiration rate (E), and nitrate in leaves were enhanced substantially in CP, while it was lower in SD. Biochemical compositions and nutrients in leaves were likewise stimulated under the culture of cocopeat-based media. Results indicate that cocopeat, sawdust, and rice husk are a possible substrates mixture in a volume ratio of 3:1:1, which would be a better choice in the cultivation of red leaf lettuce.

## 1. Introduction

Consumers are increasingly interested in balanced, fresh convenience foods, which has resulted in high demand for healthy ready-to-eat vegetables [1]. Globally, lettuce is a superior dietary fresh salad vegetable [2]. Because of the high concentrations of vitamins, minerals, dietary fiber, and antioxidant compounds, it is considered a health-promoting food [3]. Usually, plants are grown in mineral substrates such as rock wool, vermiculite, perlite, zeolite, and ceramsite culture media. Substrates come in a variety of forms, some are organic, while others are manufactured artificially [4,5]. The growing medium provides mechanical support for plants and aids in the delivery of water, nutrients, and oxygen to the roots, enabling plants to grow and develop [6]. As a result, substrate selection is one of the most important factors in soilless culture since it affects plant growth, development, and quality [7,8].

Mineral substrate like peat is more susceptible to diseases such as damping off, which can result in severe production losses [9], and their depleted lands contribute adversely and disproportionally to release accumulated carbon affecting the atmosphere and CO_2_ balance [10,11]. Thus, there is a quest for ecofriendly organic materials that can be used as alternative substrates. Soilless culture systems can be practiced by using any organic materials, such as cocopeat, rice husk, and sawdust from agricultural and agrifood waste [12,13]. Rice husk can serve as a nutrient sorbent because of its high silica (94%), carbon (37%) and ash (20%) content [14]. Sawdust is another waste product that has environmental benefits and is economically viable, so it can be used as a growing medium because it can hold moisture and nutrients [15]. Cocopeat is accepted as a growing medium with acceptable chemical properties that can be used to produce a variety of high-quality crop species [16]. This research aimed to find a suitable organic substrate combination from agro industrial wastes and assess its effectiveness as a growing media on red leaf lettuce yield and quality.

## 2. Materials and Methods

### 2.1. Plant Materials and Growing Conditions

**Figure 1:**
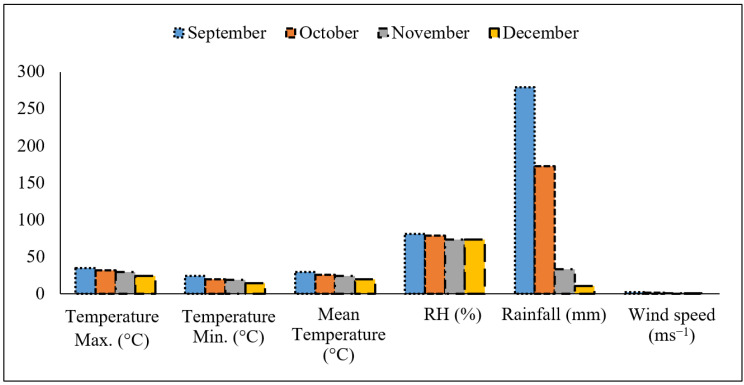
Monthly average air temperature (°C), rainfall (mm), relative humidity (%), and wind speed (ms^−1^).

The experiment was conducted in an open field at Sher-e-Bangla Agricultural University (SAU), Dhaka, Bangladesh, from September to December 2018. The experimental site is characterized by scant precipitation (Figure 1). The red lettuce (*Lactuca sativa* cv. Lollo Rossa) seeds were obtained from Japan. Seeds were surface sterilized using 70% ethanol before seeds were sown in a large plastic pot for seedling establishment. Fifteen-day-old seedlings were transferred in 15 L plastic pots containing the substrate mixtures of cocopeat: sawdust: rice husk as per treatment. The substrates were soaked overnight, washed well, and spread on a polythene sheet before filling the pots. The soaking and washing was repeated three times to remove dust particles and natural salt, which might be a hazardous factor in substrates, and soaking and washing help to moisten the substrates initially. Three plants per pot were used as an experimental unit and ~200 mL of Rahman and Inden solution [17] was added daily as a source of nutrients to the plants for 30 days. The constituents of the nutrient solution (meq L^−1^) were NO_3_-N (17.05), P (7.86), K (8.94), Ca (9.95), Mg (6.0), and S (6.0), along with the micronutrients (mg L^−1^) Fe (3.0), B (0.5), Zn (0.1), Cu (0.03), Mo (0.025), and Mn (1.0). The pH and EC of the solution were ~6.0 and 3 mS cm^−1^, respectively.

### 2.2. Experimental Design, Treatments, and Measurement of the Substrate Properties 

**Figure 2:**
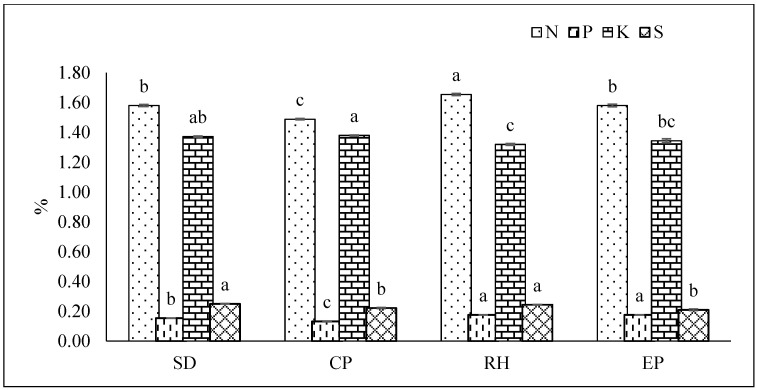
Initial nutrient composition of substrate mixtures. Abbreviations are as follows for substrate mixtures of cocopeat: sawdust: rice husk in the following proportions: 1:3:1 (SD sawdust); 3:1:1 (CP cocopeat); 1:1:3 (RH rice husk); and 1:1:1 (EP equal proportions of all three organic substrates). Different letters in the same column indicate significant differences between treatments (*p* < 0.05). Vertical bars indicate standard errors of means.

The experiment was conducted by following a randomized complete block design (RCBD) with five replications. Despite the fact that it was a pot experiment, the comparable pots were arranged in the same area of the field where the treatment combination was treated as a block. Two sets of trials were implemented independently at the same time to minimize the experimental error. Treatments were consistent with four levels of different substrate mixture, and volume ratio of the substrate was denoted as a treatment indicator. The volume ratio of cocopeat, sawdust, and rice husk was (i) SD, sawdust—1:3:1; (ii) CP, cocopeat—3:1:1; (iii) RH, rice husk—1:1:3, and (iv) EP, equal proportions of all three organic substrates—1:1:1. The properties of the substrate mixtures, viz., water holding capacity, moisture %, bulk density, air-filled porosity, pH, and electrical conductivity, and N, P, K, and S content (Table 1 and Figure 2) were measured using the following methods. Water holding capacity was measured using the formula water holding capacity (%) = {(Ws − Wd) / Wd} × 100, where Ws = weight of water-saturated substrate mixture and Wd = weight of oven-dried substrate mixture. Air-filled porosity (AFP) was determined using the formula AFP (%) = {Volume of drained water (ml) × Volume of substrate (ml)} × 100. Bulk density was determined following the core method of Teh and Talib [18]. Soil pH and electrical conductivity (EC) were measured in a water-soluble extract 1:10 (w/v) using a pH meter. Total nitrogen (N) was measured according to the Kjeldahl method [19]. The mineral contents (P, K, and S) were determined by the method reported by Mostara and Roy [19].

### 2.3. Data Collection

#### 2.3.1. Growth and Physiology

The height of the individual plant was measured with a graduated scale and the number of leaves was counted. Leaf area ratio (LAR), leaf mass ratio (LMR), shoot mass ratio (SMR), root mass ratio (RMR) were determined according to Rahman et al. [17]. The leaves, stems, and roots were separated and their corresponding fresh and dry biomass weights were measured on a sensitive balance. After immediate harvesting of leaves, the color of leaves was measured with a color spectrophotometer (NF333, Nippon Denshoku, Japan) using the CIE Laboratory L*, a*, and b* color scale. The L* value is the lightness parameter indicating the degree of lightness of the sample; it varies from 0 = black (dark) to 100 = white (light). The net photosynthetic rate (PN) and transpiration rate (E) were measured before harvesting using an ADC BioScientific LCpro portable photosynthetic system [20]. The machine was set for CO_2_: ambient, H_2_O: 0–5 mbar, photosynthetic active radiation: 1000 µmols m^−2^ sec^−1,^ and leaf temperature: 25 °C during the measurement time. The data were recorded during a sunny day between 11:00 a.m. to 2:00 p.m. The nitrate content was measured by following the standard procedure of Riga and Benedicto [21].

#### 2.3.2. Leaf Biochemical and Mineral Composition

Lettuce leaves were harvested 30 days after transplanting, when plants reached the age of 45 days following seed sowing. After immediate harvesting, fresh and dry leaves were taken as needed for evaluation of bioactive compounds and mineral nutrients. Fresh leaves were oven dried for 48 h at a temperature of around 80 °C to prepare the dry leaf samples. The leaf chlorophyll and carotenoid content were measured following the procedure given in [22]. Briefly, in a mortar, 0.2 g of fresh leaves were ground with 3 mL of acetone (80 percent v/v). The pellet was then re-extracted with 10 mL acetone solution until discoloration occurred. The solution’s upper phase was filtered, and the absorbance was taken at 470, 645, and 663 nm using a spectrophotometer. The Folin–Ciocalteu method was used to determine total phenolic content, as defined by Jayaprakasha et al. [23]. In brief, the dried leaves were ground to fine powder and the powder was dried to constant weight in desiccant at room temperature. Then, a 5 g sample was extracted with 100 mL 80% methanol for 24 h in a shaking bath. Next, 7.9 mL distilled water, 0.1 mL extract, and 0.5 mL Folin–Ciocalteu reagent (1:1 with water) were mixed in a 10 mL tube. Next, 1.5 mL sodium carbonate (10%) was added after 1 min and thoroughly mixed, and the absorbance measured at 765 nm. The total phenolic content is expressed as nmol mg^−^^1^ dry extract. Fully expanded fresh leaves (second/third from the top) were blended in mortar and pestle to extract lettuce juice, which was tested for total soluble solids, pH, and ascorbic acid. TSS (°Brix) was measured directly in the juice with a digital hand refractometer (ERMA, Tokyo, Japan) with 58–92% range, at room temperature. At room temperature, the pH of the leaf juice was determined using a digital pH meter (GroLine HI98118, Hanna, Romania) with a pH accuracy of ±0.2. The sample was placed in a 100 mL beaker, stirred, and a pH meter electrode was placed in it. Following the stabilization of the reading, direct reading from the pH meter was taken. The indophenol method outlined by Nielsen [24] was used to estimate vitamin C content. Briefly, using a mortar and pestle, 30 g of fresh leaves were ground with 10 mL of trichloroacetic acid (6%) (TCA) added. The extract was brought to 100 mL with the TCA mixture after it was ground and strained. The samples were titrated separately with the indophenol dye solution until a light rose pink color persisted for 5 s. To calculate the vitamin C content, the amount of dye used in the titration was determined. To calculate the vitamin C content, a standard curve was drawn by using ascorbic acid ranges from 10–100 mg L^−1^ and titrated, as previously described.

#### 2.3.3. Leaf Composition

Total N in leaves was investigated using the Kjeldahl method [19]. To determine the mineral content (P, K, Ca, Mg, and Fe), the lettuce leaves were dried for 72 h at 70 °C. The dry ingredients were ground and sieved through a stainless-steel sieve with a mesh size of 0.12 mm. Before weighing out 1.0 g samples for each analysis, the ground material was homogenized and re-dried for 2 h at 80 °C. The dried homogenate samples were wet digested in a 9:4:1 mixture of HNO_3_: H2SO_4_: HClO_4_, and heating was continued at 190 °C until the generation of red NO_2_ fumes stopped. The mineral content (P, K, Ca, Mg and Fe) was determined by the method reported by Mostara and Roy [19]. The spectrophotometric vanadium phosphomolybdate method was used to evaluate the P content in leaves. On a spectrophotometer, the yellow color was formed at 420 nm, and the P content from the standard curve was calculated. K, Ca, Mg and Fe estimation was performed using an atomic absorption spectrophotometer at 766.5, 422.7, 285.2, and 248.3 nm wavelength, respectively. The absorbance concentration in the sample solution represented the content of K, Ca, and Mg.

### 2.4. Statistical Analyses

Data were subjected to one-way analysis of variance to determine any significant difference in the effects of the different substrates. Statistical analysis was carried out using IBM SPSS 25.0 software (SPSS Inc, Chicago, IL, USA) to indicate the significance difference among the means by ANOVA. The mean separation was done at *p* < 0.05 using Tukey’s test.

## 3. Results

### 3.1. Substrate Characteristics

RH had the lowest water holding capacity of 68.76% while CP contained the highest at 89.50%. The water holding capacity of the rice husk-based growing media (RH) was significantly lower at 68.76% than that of SD, CP, and EP (Table 1). In contrast, the cocopeat-based media (CP) acted as an absorbent that held significantly more moisture in comparison to other substrates (Table 1). The bulk density of rice husk (0.23 g cm^−3^) was higher than cocopeat (0.19 g cm^−3^). Air-filled porosity at both 2 h and 5 h after drainage ranged from 15.24% and 17.64% in SD to 31.68% and 33.60% in RH, indicating that sawdust and cocopeat were the most efficient at retaining water in comparison to rice husks. The pH of the growing media ranged from 5.86 (EP) to 6.6 (SD), and the electrical conductivity ranged from 0.08 (SD) to 0.11 (CP). Nutrient (N, P, K, and S) availability in the growing substrates were found to vary significantly. The physical and chemical properties of the growing substrates are described in Table 1 and Figure 2.

### 3.2. Growth Characteristics

**Figure 3:**
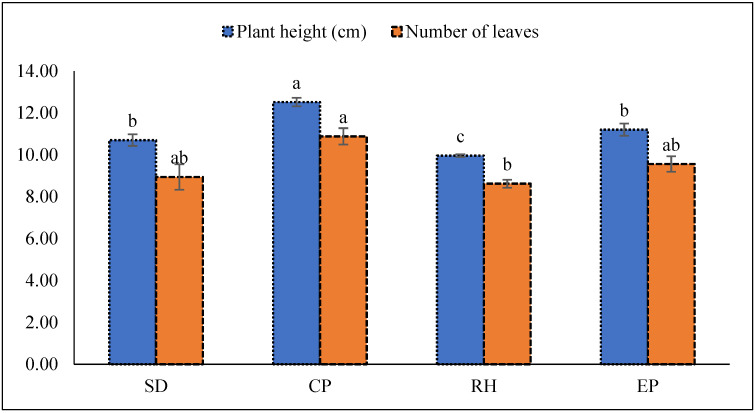
Plant height and leaf number are influenced by different growing media. Abbreviations are as follows for substrate mixtures of cocopeat: sawdust: rice husk in the following proportions: 1:3:1 (SD sawdust); 3:1:1 (CP cocopeat); 1:1:3 (RH rice husk); and 1:1:1 (EP equal proportions of all three organic substrates). Different letters in the same column indicate significant differences between treatments (*p* < 0.05). Vertical bars indicate standard errors of means.

All growing media significantly affect the plant height and leaf development, although maximum plant height and number of leaves were found for CP. Plants grown under RH were shorter and contained a fewer leaves than plants grown in CP. In contrast, a slight increase was observed under EP (Figure 3). 

Significant changes were found only in leaf area ratio by using different growing media, while there was no significant effect on leaf area, leaf area index, leaf mass ratio, shoot mass ratio, and root mass ratio (Table 2).

### 3.3. Fresh and Dry Biomass Assimilation

Fresh biomass of the whole plant, leaves, stem, and roots were significant, while fresh biomass of the shoot was insignificant. (Table 3). Cocopeat-based (CP) growing media sharply increased, measuring up to 165.93, 140.52, 13.93, and 11.48 g fresh biomass of plant, leaves, stem, and root, respectively. The plants that were grown in sawdust (SD), rice husk (RH), and an equal percentage of each medium (EP)-based growth media produced the least amount of fresh biomass (Table 3). It was perceived that CP performs best on dry biomass accumulation in the plant, including their different parts (Table 4). Plant, leaves, stem, shoot, and root dry biomass increased by 17.71%, 18.24%, 13.92%, 16.94%, and 25.97%, respectively, in cocopeat-based media, compared to sawdust-based media, which showed the lowest result. Considerably lower dry plant, leaves, stems, shoot, and root biomass growth was observed: 7.76 6.20, 0.99, 7.19, and 0.57 g, respectively (Table 4).

### 3.4. Root: Shoot

**Figure 4:**
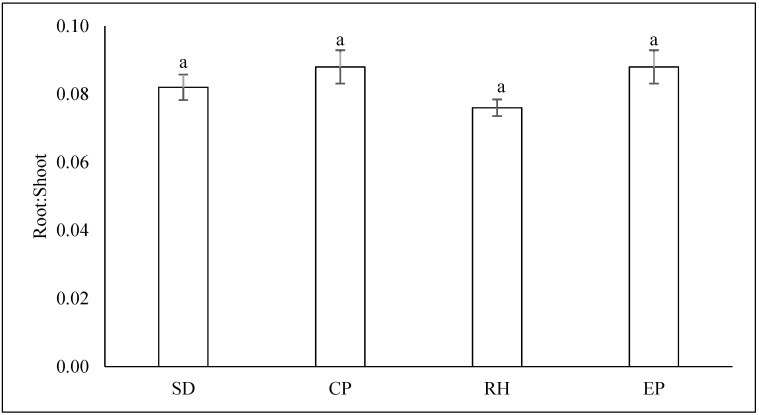
Dry root to shoot ratio of lettuce under different growing media. Abbreviations are as follows for substrate mixtures of cocopeat: sawdust: rice husk in the following proportions: 1:3:1 (SD sawdust); 3:1:1 (CP cocopeat); 1:1:3 (RH rice husk); and 1:1:1 (EP equal proportions of all three organic substrates). Different letters in the same column indicate significant differences between treatments (*p* < 0.05). Vertical bars indicate standard errors of means.

It was perceived that the growing media did not affect significantly the root to shoot ratio (Figure 4).

### 3.5. Chlorophyll and Carotenoid Content of Leaves

The cocopeat-based growing media attained the maximum value of chlorophyll a (0.51 mg g^−1^), chlorophyll b (0.15 mg g^−1^), total chlorophyll (0.66 mg g^−1^), and carotenoids (233.78 µg mg^−1^) of their fresh extract, while chlorophyll a/b (3.49) was higher in rice husk-based growing media (Table 5).

### 3.6. Leaf Color

**Figure 5:**
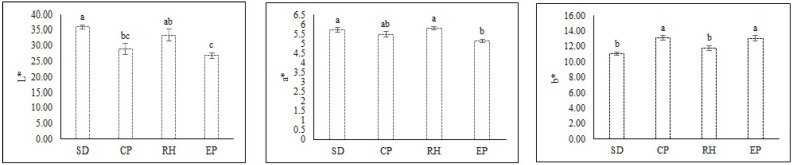
Lightness (L*), green–red chromaticity (a*), and blue–yellow chromaticity (b*) of lettuce leaf under different growing media cultures. Abbreviations are as follows for substrate mixtures of cocopeat: sawdust: rice husk in the following proportions: 1:3:1 (SD sawdust); 3:1:1 (CP cocopeat); 1:1:3 (RH rice husk); and 1:1:1 (EP equal proportions of all three organic substrates). Different letters in the same column indicate significant differences between treatments (*p* < 0.05). Values are mean ± SE.

With the different growing media, the lettuce leaf exhibited maximum L values (35.94) for SD, a* values (5.80) for RH, and b* values (13.10) for CP, in comparison to all respective media (Figure 5). The value (L* < 50) indicated that leaves showed extreme darkening. All of the leaves tended towards the positive values of redness parameter (a*) of leaf color, indicating that there was less or no excess browning. In addition, all of the leaves tended toward yellow as indicated by positive values of yellowness (b*) parameter (Figure 5).

### 3.7. Net Photosynthetic and Transpiration Rate in Leaves

PN and E in leaves were enhanced due to growing media mixtures. Lettuce leaves attained the maximum values of PN (17.28 µmol CO_2_ m^−2^ s^−1^) and E (6.14 mmol m^−2^ s^−1^) for CP, while the plants growing in SD (13.42 µmol CO_2_ m^−2^ s^−1^ and 5.94 mmol m^−2^ s^−1^ for PN and E, respectively) gave the lowest value (Table 6). 

### 3.8. Biochemical Compounds in Leaves

Total phenol content, TSS, leaf juice pH, vitamin C, and nitrate content significantly increased under different growing media (Table 7) treated with Rahman and Inden solution [17]. The plants grown in CP gave the highest value of these parameters, except for TSS and leaf juice pH (SD), in comparison with other growing media. 

### 3.9. Mineral Nutrients in a Lettuce Leaf

The value relating to mineral nutrients composition in lettuce leaves (Table 8) clearly showed that growing media had a significant effect on Ca, Mg, N, P, K, and Fe. The maximum mean value of this parameter was attained in cocopeat-based media (CP) compared to other media. 

## 4. Discussion

Unlike a usual soil profile, a plastic pot atmosphere affords a quite shallow growing substrate layer that becomes saturated swiftly by watering. The increase of cocopeat in the mixture increased the water holding capacity because it acts as an absorbent that can hold a large volume of water. Note that the capacity to hold water is reflected in the different organic media [15,25]. The bulk density of the two substrates could be due to the difference in particle size.

In air-filled porosity at two and five hours after drainage, after saturation conditions, water was easily drained by gravitational forces from RH and EP (Table 1). This could be due to the particle size and volume of large pores in these substrates. In essence, an active growing medium must have a physical structure capable of maintaining a desirable balance between air and water storage for porosity during and between irrigation processing [26].

In a hydroponic system, a pH of 8.4–8.8 is higher than recommended for growing media, which can have a negative effect on plant growth [27]. In terms of EC, less than 3.5 dSm^–1^ is considered the limit for seedling growth in a growing medium [28], whereas an EC of more than 4 dSm^−1^ has been shown to inhibit seed germination [29]. In this study, pH and EC (Table 1) of the growing substrates were within the acceptance ranges for the production of lettuce in containers. This may be due to the anoxic conditions arising from slow drainage and higher capacity to hold water (Table 1), which makes plant nutrients accessible [15]. Ion concentration in the media depends primarily on EC values controlling the availability of nutrients [16]. As a result, neither pH nor EC had any negative effects on plant development. These findings are consistent with Morales et al. [30] and Giménez et al. [31].

Plant growth, leaf composition, total yield, and fruit quality are affected by the substrates in soilless culture [32,33]. Plant growth is aided by the growing medium, which may be attributed to the media’s availability of nutrients. According to Trevisan et al. [34], organic media such as compost serves as a nutrient buffer, slowly releasing nutrients to plant roots [35]. The increased plant height and number of leaves observed in the experiment (Figure 3) is due to cocopeat having a high water holding capacity, aeration, EC with low bulk density association, and a strong nutrient supply with EC acceptance rate over the growing period (Table 1). Cocopeat has a maximum capacity for water retention, aeration, and EC, and provides a good nutrient source for growing bitter gourds [36]. Results showed that RH retained high pore spaces and bulk density, which could adversely affect plant growth. The experimental media, however, provide a slightly higher pH that can be minimized with acid-based fertilizer and tolerated by plants [25].

The plant’s vegetative growth, and fresh biomass (Table 3), is associated with food stored either in leaves, shoots, or roots. Higher capacity for holding water, better aeration with lower bulk density, and EC of growing media help to maintain a satisfactory atmosphere that resulted in vigorous plant growth, which ensures increased photosynthetic potential by leaves. Water retention ability, gaseous exchange, and root penetration depend on the amount of pore space in the media, which helps to improve plant growth [17,37]. Dry plant biomass (Table 4) may also rely on photosynthates gathered in leaves that have beneficially influenced the accumulation of dry matter in lettuce plants.

The amount of chlorophyll and carotenoids in vegetables varies depending on the growing conditions [38,39]. The development of leaf chlorophyll and carotenoids (Table 4) relies on the accumulation of nitrogen in plants under organic substrates, which increase aeration, water holding capacity, and biostability. Organic substrates that have good aeration, water holding capability, and biostability lead to absorption of N for the chlorophyll development in bitter gourd leaves [36]. Plant growth and development were hindered, resulting in lower chlorophyll content in leaves, except for cocopeat, under substrate culture. The likely cause behind this outcome was the availability of nutrients in this media [36,40].

All the leaves could be linked to yellowness (b*) positive values (Figure 5) due to antioxidant content and enzyme activity, which could also be controlled by various growing media with nutrient solution. The color of fruit, flesh, and placenta in bitter gourd depends on the balance of nutrients in various growing mediums [36], while the flesh color of potato varieties varies as a result of low reducing sugars [41].

Organic growing media mixtures play an important role in the growth and development of plants [36]. When lettuce is grown in CP, the content of PN and E in leaves is enhanced (Table 6). This may be due to adequate aeration, water resistance, lower bulk density, and increased media biostability, which in terms of lettuce production provide adequate nutrients compared to other media. As suggested by Christoulaki et al. [42], high sawdust content (75–100%) in substrates reduced leaf photosynthetic rates and stomatal conductance with minimal changes in intercellular CO_2_ concentration, and this fluctuation occurs due to changes in the substrate physicochemical properties (aeration, water holding capacity, etc.) during plant development.

The rise in biochemical compounds in leaves (Table 7) may be due to the substrate physical properties, which induces beneficial microbial activity and stimulates the process of photosynthesis (Table 6). The total phenolic content of lettuce grown in cocopeat-based growing media was higher than that of plants grown in other media used in this study (Table 7). The nature of the growing media, season, and root zone temperature (not mentioned) may all be factors in the higher phenolic compound in lettuce leaves. According to Lakhdar et al. [43], compounds rich in the ability to cause an oxidative process in plants can be found in the compost made from tomato, leek, vineyard, and olive mill cake residues used as growing media. These compounds stimulated the secondary metabolites in lettuce grown in various agroindustrial byproducts [31,44]. In greenhouse cultivation, temperature rises in the root zone of leafy vegetables can cause changes in the development of secondary metabolites, primarily phenolic compounds [45,46]. Plant growth in CP depends largely on the media physical properties, making it easier to obtain higher mineral elements under a balanced nutrient environment that eventually leads to higher biochemical activity [36,40]. According to Jankauskien et al. [47], growing tomatoes in a coconut substrate changed the biochemical composition of the fruits, resulting in more sugar, less ascorbic acid, and less lycopene in the fruits [48]. Sugar, soluble solids, and pH levels in lettuce leaves grown in coconut fiber were found to be within acceptable limits in this study (Table 7).

Baby leaf vegetables are a significant source of nitrates [1]; thus, the nitrate content is an essential quality characteristic to consider. Plants grown in CP had higher nitrate content than the other substrates. This may be attributed to improvements in the nutrient solution, and a gradual release of nitrate from the media as a result of increased nitrogen mineralization [49,50].

The type of substrate has an effect not only on plant yield, but also on quality profile [48,51,52]. The nutrient balance in the leaves is influenced by textured rice husk and cocopeat, and is regulated by the nutrient solution by maintaining proper humidity and temperature levels [36]. In this study, mineral nutrients in lettuce leaves (Table 8) shows quite clearly that CP had a significant effect on the accumulation of Ca, Mg, N, P, K, and Fe. Plants grown in substrate mixtures with higher sawdust content had no significant effects on elemental uptake [42].

## 5. Conclusions

The results of this study showed that adding cocopeat to other organic growing media can enhance certain chemical and physical properties. With cocopeat-based media, plant growth, and physiological, biochemical, and balanced accumulation of mineral nutrients in leaves is significantly enhanced. Often influenced by substrates are lightness chromaticity (L*), blue–yellow chromaticity (b*), and green–red chromaticity (a*) of lettuce leaves. Cocopeat-based media increases lettuce quality by increasing total phenolic content and nutrient compositions (Ca, Mg, N, P, K, and Fe), and improving lettuce growth by acting as a biofertilizer. In contrast to the other treatments, cocopeat-based media had higher amounts of nitrate, chlorophyll, carotenoids, and vitamin C in lettuce leaves. Cocopeat-based mixtures enhanced the amount of photosynthetic rate (PN) and transpiration rate (E) in leaves relative to other substrates. Taking into account the effect of all growing media mixture, cocopeat-based media practice had a potential positive impact on lettuce culture. To grow red leaf lettuce in an aggregate hydroponic system, a cocopeat-based media mixture with balanced nutrient management could be the adjuvant.

## Figures and Tables

**Table 1:** Physical and chemical properties of substrate mixtures.

Substrates	Water Holding Capacity (%)	Moisture (%)	Bulk Density (g cm^−3^)	Air-Filled Porosity (%) after 2 h	Air-Filled Porosity (%) after 5 h	pH	Electrical Conductivity (dS m^−1^)
SD	72.53 ± 0.76 ^c^	83.00 ± 0.32 ^b^	0.21 ± 0.00 ^b^	15.24 ± 0.19 ^d^	17.64 ± 0.39 ^d^	6.60 ± 0.04 ^a^	0.08 ± 0.00 ^b^
CP	89.50 ± 0.24 ^a^	84.33 ± 0.37 ^a^	0.19 ± 0.00 ^c^	22.63 ± 0.39 ^c^	24.86 ± 0.72 ^c^	6.34 ± 0.07 ^ab^	0.11 ± 0.00 ^a^
RH	68.76 ± 0.52 ^d^	82.81 ± 0.16 ^b^	0.23 ± 0.00 ^a^	31.68 ± 0.42 ^a^	33.60 ± 0.51 ^a^	6.12 ± 0.04 ^ab^	0.11 ± 0.01 ^a^
EP	76.28 ± 0.71 ^b^	82.80 ± 0.20 ^b^	0.20 ± 0.01 ^bc^	25.48 ± 0.22 ^b^	27.84 ± 0.48 ^b^	5.86 ± 0.19 ^b^	0.10 ± 0.00 ^ab^
F-value	233.05	5.76	20.44	358.72	147.83	7.50	7.44
*p*-value	0.00	0.01	0.00	0.00	0.00	0.00	0.00

Abbreviations are as follows for substrate mixtures of cocopeat: sawdust: rice husk in the following proportions: 1:3:1 (SD sawdust); 3:1:1 (CP cocopeat); 1:1:3 (RH rice husk); and 1:1:1 (EP equal proportions of all three organic substrates). Different letters in the same column indicate significant differences between treatments (*p* < 0.05). Values are mean ± SE.

**Table 2:** Leaf, stem, and root growth features of lettuce in soilless culture.

Substrates	Leaf Area (cm^2^)	Leaf Area Index	Leaf Area Ratio	Leaf Mass Ratio	Stem Mass Ratio	Root Mass Ratio
SD	117.24 ± 1.74 ^a^	1.17 ± 0.02 ^a^	15.12 ± 0.36 ^a^	0.80 ± 0.01 ^a^	0.13 ± 0.01 ^a^	0.07 ± 0.00 ^a^
CP	119.08 ± 2.10 ^a^	1.19 ± 0.02 ^a^	12.65 ± 0.36 ^b^	0.80 ± 0.01 ^a^	0.12 ± 0.01 ^a^	0.08 ± 0.00 ^a^
RH	115.96 ± 2.31 ^a^	1.16 ± 0.02 ^a^	14.48 ± 0.67 ^a^	0.81 ± 0.01 ^a^	0.11 ± 0.01 ^a^	0.07 ± 0.00 ^a^
EP	117.04 ± 2.18 ^a^	1.17 ± 0.02 ^a^	14.60 ± 0.32 ^a^	0.79 ± 0.00 ^a^	0.13 ± 0.00 ^a^	0.08 ± 0.00 ^a^
F-value	1.56	1.57	5.74	1.97	0.74	1.91
*p*-value	0.25	0.25	0.01	0.16	0.54	0.17

Abbreviations are as follows for substrate mixtures of cocopeat: sawdust: rice husk in the following proportions: 1:3:1 (SD sawdust); 3:1:1 (CP cocopeat); 1:1:3 (RH rice husk); and 1:1:1 (EP equal proportions of all three organic substrates). Different letters in the same column indicate significant differences between treatments (*p* < 0.05). Values are mean ± SE.

**Table 3:** Fresh biomass (g) assimilation in plant, leaves, stem, shoot, and root influenced by different growing media.

Substrates	Total Plant	Leaf	Stem	Shoot	Root
SD	153.60 ± 2.01 ^b^	130.95 ± 1.61 ^b^	12.56 ± 0.27 ^b^	143.51 ± 1.79 ^a^	10.08 ± 0.37 ^b^
CP	165.93 ± 3.61 ^a^	140.52 ± 3.46 ^a^	13.93 ± 0.30 ^a^	154.45 ± 3.58 ^a^	11.48 ± 0.26 ^a^
RH	157.89 ± 2.69 ^ab^	134.59 ± 2.73 ^ab^	12.94 ± 0.07 ^b^	147.53 ± 2.74 ^a^	10.35 ± 0.22 ^b^
EP	162.84 ± 2.76 ^ab^	139.08 ± 2.67 ^ab^	13.08 ± 0.19 ^b^	152.16 ± 2.71 ^a^	10.68 ± 0.19 ^ab^
F-value	5.11	3.81	12.04	3.08	6.56
*p*-value	0.02	0.04	0.00	0.06	0.01

Abbreviations are as follows for substrate mixtures of cocopeat: sawdust: rice husk in the following proportions: 1:3:1 (SD sawdust); 3:1:1 (CP cocopeat); 1:1:3 (RH rice husk); and 1:1:1 (EP equal proportions of all three organic substrates). Different letters in the same column indicate significant differences between treatments (*p* < 0.05). Values are mean ± SE.

**Table 4:** Dry biomass (g) assimilation in plant, leaves, stem, shoot, and root influenced by different growing media.

Substrates	Total Plant	Leaf	Stem	Shoot	Root
SD	7.76 ± 0.09 ^b^	6.20 ± 0.10 ^b^	0.99 ± 0.06 ^ab^	7.19 ± 0.09 ^b^	0.57 ± 0.02 ^b^
CP	9.43 ± 0.19 ^a^	7.51 ± 0.21 ^a^	1.15 ± 0.04 ^a^	8.66 ± 0.20 ^a^	0.77 ± 0.04 ^a^
RH	8.05 ± 0.21 ^b^	6.56 ± 0.19 ^b^	0.91 ± 0.06 ^b^	7.47 ± 0.21 ^b^	0.58 ± 0.02 ^b^
EP	8.04 ± 0.26 ^b^	6.36 ± 0.19 ^b^	1.02 ± 0.05 ^ab^	7.38 ± 0.23 ^b^	0.66 ± 0.04 ^ab^
F-value	14.34	11.04	3.56	12.32	8.78
*p*-value	0.00	0.00	0.04	0.00	0.00

Abbreviations are as follows for substrate mixtures of cocopeat: sawdust: rice husk in the following proportions: 1:3:1 (SD sawdust); 3:1:1 (CP cocopeat); 1:1:3 (RH rice husk); and 1:1:1 (EP equal proportions of all three organic substrates). Different letters in the same column indicate significant differences between treatments (*p* < 0.05). Values are mean ± SE.

**Table 5:** Chlorophyll and carotenoid content of leaves under different growing media cultures in lettuce.

Substrates	Chlorophyll a	Chlorophyll b	Total Chlorophyll	Chlorophyll a/b	Carotenoids
SD	0.47 ± 0.008 ^b^	0.14 ± 0.000 ^b^	0.61 ± 0.008 ^b^	3.36 ± 0.059 ^ab^	227.18 ± 0.77 ^b^
CP	0.51 ± 0.008 ^a^	0.15 ± 0.003 ^a^	0.66 ± 0.010 ^a^	3.42 ± 0.046 ^ab^	233.78 ± 0.67 ^a^
RH	0.47 ± 0.008 ^b^	0.14 ± 0.002 ^b^	0.61 ± 0.006 ^b^	3.49 ± 0.118 ^a^	223.74 ± 0.35 ^c^
EP	0.46 ± 0.002 ^b^	0.15 ± 0.000 ^a^	0.61 ± 0.002 ^b^	3.09 ± 0.014 ^b^	228.72 ± 0.66 ^b^
F-value	8.43	15.89	12.76	5.11	55.46
*p*-value	0.00	0.00	0.00	0.01	0.00

Chlorophylls are expressed as mg g^−^^1^ fresh extract and carotenoids as µg mg^−^^1^ fresh extract. Abbreviations are as follows for substrate mixtures of cocopeat: sawdust: rice husk in the following proportions: 1:3:1 (SD sawdust); 3:1:1 (CP cocopeat); 1:1:3 (RH rice husk); and 1:1:1 (EP equal proportions of all three organic substrates). Different letters in the same column indicate significant differences between treatments (*p* < 0.05). Vertical bars indicate standard errors of means.

**Table 6:** Net photosynthetic and transpiration rate in lettuce leaf under different growing media.

Substrates	Net Photosynthetic Rate (P_N_)	Transpiration Rate (E)
SD	13.42 ± 0.16 ^d^	5.94 ± 0.04 ^b^
CP	17.28 ± 0.12 ^a^	6.14 ± 0.07 ^a^
RH	15.20 ± 0.12 ^c^	6.04 ± 0.04 ^ab^
EP	16.60 ± 0.20 ^b^	6.06 ± 0.02 ^ab^
F-value	112.24	3.22
*p*-value	0.00	0.05

Net photosynthetic rate (P_N_**)** is expressed as µmol CO_2_ m^−2^ s^−1^ and transpiration rate (E) as mmol m^−2^ s^−1^. Abbreviations are as follows for substrate mixtures of cocopeat: sawdust: rice husk in the following proportions: 1:3:1 (SD sawdust); 3:1:1 (CP cocopeat); 1:1:3 (RH rice husk); and 1:1:1 (EP equal proportions of all three organic substrates). Different letters in the same column indicate significant differences between treatments (*p* < 0.05). Values are mean ± SE.

**Table 7:** Biochemical composition in lettuce leaves under different growing media culture.

Substrates	Total Phenolic Content	TSS	Leaf Juice pH	Vitamin C	Nitrate Content
SD	131.28 ± 0.46 ^c^	5.90 ± 0.10 ^a^	6.38 ± 0.10 ^a^	458.65 ± 1.19 ^b^	257.06 ± 0.95 ^b^
CP	136.32 ± 0.50 ^a^	5.78 ± 0.12 ^ab^	6.04 ± 0.04 ^b^	473.94 ± 2.51 ^a^	267.48 ± 2.48 ^a^
RH	133.78 ± 1.01 ^bc^	5.44 ± 0.12 ^b^	6.12 ± 0.07 ^ab^	464.46 ± 1.79 ^b^	263.04 ± 1.46 ^ab^
EP	135.26 ± 0.23 ^ab^	5.88 ± 0.10 ^a^	6.04 ± 0.02 ^b^	463.00 ± 1.38 ^b^	266.80 ± 1.24 ^a^
F-value	12.48	3.92	5.77	19.88	7.96
*p*-value	0.00	0.03	0.01	0.00	0.00

Total phenolic content is expressed as nmol mg^−1^ dry extract, TSS as %, vitamin C and nitrate content as mg kg^−1^ fresh extract. Abbreviations are as follows for substrate mixtures of cocopeat: sawdust: rice husk in the following proportions: 1:3:1 (SD sawdust); 3:1:1 (CP cocopeat); 1:1:3 (RH rice husk); and 1:1:1 (EP equal proportions of all three organic substrates). Different letters in the same column indicate significant differences between treatments (*p* < 0.05). Values are mean ± SE.

**Table 8:** Nutrient balance in lettuce leaf under different growing substrate culture.

Substrates	Ca	Mg	N	P	K	Fe
SD	8.76 ± 0.21 ^b^	3.44 ± 0.09 ^b^	41.05 ± 0.32 ^bc^	4.10 ± 0.03 ^b^	70.49 ± 0.44 ^b^	82.90 ± 0.13 ^c^
CP	10.90 ± 0.26 ^a^	3.78 ± 0.07 ^a^	43.03 ± 0.45 ^a^	4.22 ± 0.02 ^a^	73.34 ± 0.67 ^a^	85.69 ± 0.18 ^a^
RH	9.34 ± 0.19 ^b^	3.16 ± 0.09 ^b^	40.05 ± 0.28 ^c^	4.14 ± 0.04 ^ab^	71.26 ± 0.55 ^ab^	83.57 ± 0.21 ^bc^
EP	10.36 ± 0.18 ^a^	3.50 ± 0.04 ^ab^	42.00 ± 0.32 ^ab^	4.16 ± 0.02 ^ab^	72.60 ± 0.40 ^ab^	84.08 ± 0.24 ^b^
F-value	16.42	9.87	13.34	3.46	6.00	55.34
*p*-value	0.00	0.00	0.00	0.04	0.01	0.00

Mineral nutrients are expressed as mg kg^−1^ dry extract. Abbreviations are as follows for substrate mixtures of cocopeat: sawdust: rice husk in the following proportions: 1:3:1 (SD sawdust); 3:1:1 (CP cocopeat); 1:1:3 (RH rice husk); and 1:1:1 (EP equal proportions of all three organic substrates). Different letters in the same column indicate significant differences between treatments (*p* < 0.05). Values are mean ± SE.
